# Maternal characteristics and nutritional status among 6–59 months of children in Ethiopia: further analysis of demographic and health survey

**DOI:** 10.1186/s12887-019-1459-x

**Published:** 2019-03-20

**Authors:** Zufan Bitew Dessie, Melkitu Fentie, Zegeye Abebe, Tadesse Awoke Ayele, Kindie Fentahun Muchie

**Affiliations:** 1 Central Gondar Zone Health Department, Amhara Regional State, AmbaGiorgis, Ethiopia; 2 Department of Human Nutrition, Institute of Public Health, College of Medicine and Health Sciences, University of Gondar, Gondar, Ethiopia; 3 Department of Epidemiology and Biostatistics, Institute of Public Health, College of Medicine and Health Sciences, University of Gondar, Gondar, Ethiopia; 4 Department of Epidemiology and Biostatistics, School of Public Health, College of Medicine and Health Sciences, Bahir Dar University, Bahir Dar, Ethiopia

**Keywords:** Maternal characteristics, Stunting, Wasting, Children, Ethiopia, DHS, GEE

## Abstract

**Background:**

Nutritional status of children influences their health status, which is a key determinant of human development. In Ethiopia, 28% of child mortality is caused by under nutrition. There is also some controversial evidence about the association between maternal characteristics and nutritional status of under five children. This study was aimed to assess the association between maternal characteristics and nutritional status among 6–59 months of children in Ethiopia.

**Methods:**

This was furtheranalysis ofthe 2016 Ethiopian Demographic and Health Surveyusing7452 children.. Generalized estimating equations was used to quantify the association of maternal factors with stunting and wasting. Both crude Odds ratio and adjusted odds ratio with the corresponding 95% confidence intervals were reported to show the strength of association. In multivariable analysis, variables with a *p*-value of < 0.05 were considered statistically significant.

**Results:**

The higher odds of stunting were found among children whose mothers had no education (AOR = 1.58; 95%CI: 1.25, 2.0) and primary education (AOR = 1.42; 95%CI: 1.13, 1.78), underweight nutritional status (AOR = 1.59; 95%CI: 1.27, 2.0), and anemia (AOR = 1.16; 95%CI: 1.04, 1.30). Similarly, higher odds of wasting were observed among children whose mother had underweight nutritional status (AOR = 2.34; 95%CI: 1.65, 3.38), delivered at home (AOR = 1.31; 95%CI: 1.07, 1.60), and lower than 24 months birth interval (AOR = 1.31; 95%CI: 1.04, 1.64).

**Conclusion:**

Maternal education, nutritional status, and anemia were associated with child stunting. Also maternal nutritional status, place of delivery, and preceding birth interval were associated with wasting. Therefore, there is needed to enhance the nutritional status of children by improving maternal underweight nutritional status, maternal educational and maternal anemia status, prolonging birth interval, and promoting health facility delivery.

## Background

Malnutrition refers to imbalances in intake of energy, protein and or other nutrients and encompass both under and over-nutrition. Under nutrition, a group of disorders that includes stunting, wasting and underweight. It is the result of inadequate intake of food, infection, inadequate access to food, inadequate care and feeding practices, limited health services and unhealthy environment and poor financial, human physical and social capital [1–4]. It is recognized as a public health problem especially in developing country, mostly affecting children, women of childbearing age and pregnancy.

Child under nutrition has long-term negative effects on individuals and communities in all areas of life, including health, education, and productivity and seriously affects the human capital of a country on which the economy relies. It is due to undernutrition is strongly associated with faltered growth, delayed mental development, and reduced intellectual capacity [5, 6]. In addition, it has been associated with overweight, obesity, insulin resistance, and chronic non-communicable disease during adulthood [7].

Globally, under nutrition in children is highly prevalent and remains a big challenge [1]. According to United Nations Children’s Fund (UNICEF) report, 25% of children under the age of five years are stunted, 16% underweight and 8% wasted, and an estimated 6.3 million live born children worldwide died before age 5 years, because of under nutrition [8]. In addition, nearly half of all deaths in children under 5 are attributable by under nutrition. The highest prevalence of under-five under nutrition is found in Africa (36%) followed by Asia (27%). Accordingly, the three forms of malnutrition in Sub-Saharan Africa was 40, 21 and 9% stunting, underweight and wasting, respectively [9, 10].

In Ethiopia, among children under age five, 38% were stunted, 24% are underweight, and 10% are wasted [11]. The burden of malnutrition in Ethiopia is the second highest in SSA [4]. Ethiopia has made progress in reducing hunger and, to an extent, under nutrition; however, malnutrition is one of the major public health problem in Ethiopia [1]. Accordingly, the country endorsed a National Nutrition Program, prepared infant and young child feeding manual, and implemented monthly child growth and monitoring program. However, it is remained as a major public health problem in Ethiopia.

Child under nutrition in Ethiopia is the result of several complex, multidimensional, and interrelated factors that operate at different levels from which maternal characteristic is one of the factors. As mothers are the main providers of primary care to their children, understanding the contribution of maternal characteristics on child nutrition is a key towards addressing the problem of child under nutrition [1, 12]. Consequently, understanding of maternal characteristics could be important to achieve the country plan of ending childhood under nutrition by 2030.

In Ethiopia, most studies on child nutrition were descriptive and few have been conducted analytically but were founded on pocket area survey data that might be difficult to generalize diverse Ethiopia.

Therefore, we analyzed EDHS data to assess the association between maternal characteristics and nutritional status among 6–59 months of children in Ethiopia. The result of this analysis will generate evidence for policy makers and program designer to take appropriate action to achieve the national goal of reaching zero level of childhood under nutrition by 2030.

## Methods

### Study area and design

The study was conducted in Ethiopia, is found in the East Horn of Africa at the Sub Sahara. In Ethiopia, as in most African countries, women play the principal roles in the rearing of children and the management of family affairs. On the other hand, the health status of these women remains poor.

This was a further analysis of the 2016 Ethiopian Demographic and Health Survey (EDHS) conducted from January 18,2016 to June 27, 2016 [11]. The 2016 EDHS was a national representative cross-sectional survey and it is part of the worldwide MEASURE DHS project which was implemented by the Ethiopian Central Statistical Agency (CSA).

### Study population

The 2016 EDHS is the fourth survey conducted in nine regional states namely; Tigray, Afar, Amhara, Oromia, Somali, Benishangul Gumuz, Southern Nations Nationalities and Peoples (SNNP), Gambella and Harari and two city Administrations namely; Addis Ababa and Dire Dawa. The target population was all 6–59 months of children in Ethiopia. Whereas, study population were all 6–59 months of children in the randomly selected enumeration areas (EAs) of Ethiopia.

### Sampling procedures

The 2016 EDHS use a stratified, two-stage cluster sampling to identify the representative samples. The sampling frame for the 2016 EDHS consists of a total of 84,915 Enumeration Areas (EAs). On the first stage645 EAs (202 in urban areas and 443 in rural areas) were selected. Then, on the second stage, a fixed number of 28 households are selected from each EA. A total of 16,650 households are included in the interview. The survey interviewed a nationally representative population of 9504 children age 6–59 months in the selected households, of which 7452children with complete anthropometric record had included in this analysis. The detailed explanation of sampling procedure can be found in the methodology of the EDHS final report [11].

### Data collection procedures

The EDHS used structured and pre-tested questionnaire as a tool for data collection. Structured interview schedules were performed by trained interviewers. Frequent supervision was performed during data collection and interviews were performed using local languages. Socioeconomic; and demographic, child and maternal characteristics related information was collected from child, women and household questionnaires.

Height and weight measurements were carried out on under-5 children in all selected households. Weight measurements were obtained using lightweight SECA mother-infant scales with a digital screen designed and manufactured under the guidance of UNICEF. Children younger than 24 months were measured for height while lying down, and older children were measured while standing using a Shorr measuring board. The detailed explanation of data collection procedure can be found in the methodology of the EDHS final report [11].

### Variables of the study

The outcome variables of this study were Stunting and wasting. Accordingly, for stunting,0 coding implies ‘not stunted’ and 1 coding implies stunted [13]. And also Wasting was coded as 0for ‘not wasted’ and 1 coding implies wasted [13].

Independent variables were selected based on literatures [12] and their availability in our data. They were socio-demographic characteristics and environmental factors such asage of child, sex of child, age of mother, level of education, employment status, wealth status, residence, marital status, region, family size, source of drinking water, type of toilet facility. Also, maternal characteristics such as preceding birth to conception interval, antenatal care visit, anemia status, nutritional status, place of delivery. Additionally, we included child factors such assize at birth, child’s health status (fever, diarrhea, and cough), breast feeding status, dietary diversity, and birth order.

Maternal nutritional status was classified as underweight (≤18.4); normal (18.5–24.9); overweight (25.0–29.9); obese (≥30 kg/m^2^) using BMI (weight (kg)/height (m^2^)) according to the definitions of the World Health Organization [14]. Besides, maternal anemia status was classified as anemic if Hb ≤ 11.0 g/dl [15].

Minimum dietary diversity score (DDS) was assessed using 24 h dietary recall method based on seven food groups in the local context. Then the reported food items were classified in to grains/roots/tubers; legumes and nuts; dairy products; flesh foods (meats/fish/poultry); eggs; vitamin A-rich fruits and vegetables; and other fruits and vegetables. Then those children who consumed four or more food groups out of the seven food groups were defined as having adequate dietary diversity score [13]. Early initiation of breastfeeding, exclusive breastfeeding, and ever breastfeeding status of the child was collected from the mother/caregivers and the detail procedure is found from EDHS 2016 report.

### Data processing and analysis

The data were cleaned and analyzed using STATA version 14 Software. Descriptive analyses were conducted to describe the characteristics of the study participants and the result was presented using text and table. Generalized estimating equations (GEE) with binomial family and exchangeable correlation structure, were used to determine association of maternal factors with stunting and wasting in a child. GEE adjusts the standard errors by accounting clustered observations. An exchangeable correlation structure was chosen for the main models by assuming two observations are equally correlated within a cluster, with no correlation between observations from different clusters. Accordingly, the bi-variable GEE for maternal characteristics, child factors, environmental factors and socio demographic factors on child stunting and wasting were fitted. All variables with *p*-value ≤0.2 in the bi-variable were fitted in multivariable GEE. Both crude odds ratio (COR) and adjusted odds ratio (AOR) with the corresponding 95% confidence intervals were reported to show the strength of association. In multivariable analysis, variables with a p-value of < 0.05 were considered statistically significant.

## Results

### Socio-demographic and economic characteristics

A total of 7452 children aged 6–59 months were included in the analysis. Among the total children, 3816 (51.2%) were males, and 1692(22.7%) found in the age group of 12–23 months. The mean (±SD) age of the child was 31.63(±15.62) months. About 3650 (49%) children were lived-in a family size of 5–7. Most, 6161 (82.7%), of children were lived in rural areas. About 5419 (72.7%) children of mothers were in the age range of 20–34 years and 6963 (93.4%) mothers of children were married. About 4823 (64.7%) and 5334(71.6%) mothers of children were uneducated and unemployed, respectively (Table 1).

**Table 1:** Socio-demographic and economic characteristics of children (6–59 months) and their mother in Ethiopia, May 2018 (*n* = 7452)

Variables	Frequency	Percentage
Age of child in months		
6–11	887	11.9
12–23	1692	22.7
24–35	1620	21.7
36–47	1584	21.3
48–59	1669	22.4
Age of mother		
15–19	221	3.0
20–34	5419	72.7
35–49	1812	24.3
Region		
Tigray	813	10.9
Afar	700	9.4
Amhara	771	10.3
Oromia	1171	15.7
Somali	939	12.6
Benishangul	634	8.5
SNNPR	958	12.9
Gambela	477	6.4
Harari	343	4.6
Dire Dawa	343	4.6
Addis Ababa	303	4.1
Mother’s educational level		
No education	4823	64.7
Primary	1915	25.7
Secondary /above	714	9.6
Mother’s Employment status		
Not employed	5334	71.6
Employed	2118	28.4
Family size		
2–4	2009	27.0
5–7	3650	49.0
> =8	1793	24.1
Source of drinking water		
Improved water source*	4472	60.0
Unimproved water source^#^	2980	40.0
Type of toilet facility		
Improved toilet**	1186	15.9
Unimproved toilet***	3051	40.9
Open defecation	3215	43.1
Wealth status		
Poor	4002	53.7
Medium	1121	15.0
Rich	2329	31.3

*piped water, public tap/stand pipe, tube well or bore hole, protected dug well, protected spring, rain water, bottled water ^#^unprotected dug well, unprotected spring, tanker truck/cart with small tank, surface water **flush toilet system, ventilated improved pit latrine, pit latrine with slab, composting toilet ***pit latrine without slab/open pit, bucket toilet, hanging toilet, flush not to piped sewer

### Child and maternal characteristics

About 1944 (26.1%) of the children were small sized at birth. Nearly one-third, 2391 (32.1%), had 2–3 birth order and 7149 (95.9%) children were breast feed. A total of 583 (13%) children had adequate dietary diversity. Among the participants 899 (12.1%), 1104 (14.8%), 1267 (17%) of children had experienced diarrhea, fever and cough in the last two weeks preceding the date of survey, respectively (Table 2).

**Table 2:** Child and maternal characteristics of children aged 6–59 months in Ethiopia, May 2018 (*n* = 7452)

Variables	Frequency	Percentage
Size of child at birth		
Large	2255	30.3
Average	3253	43.7
Small	1944	26.1
Birth order number		
1	1443	19.4
2–3	2391	32.1
4–5	1751	23.5
> =6	1867	25.1
Breast feeding status		
Breast feed	7149	95.9
Never breast feed	303	4.1
Dietary diversity		
Inadequate	3906	87
Adequate	583	13
Mother’s nutritional status		
Under weight	1845	24.8
Normal	4967	66.7
Over weight/ Obese	640	8.6
Preceding birth interval		
First birth	1443	19.4
< 24	1515	20.3
24–47	3084	41.4
> =48	1410	18.9

About 1845 (24.8%) mothers of children were underweight. Around two-third, 3316 (67.1%), mothers of children had ANC follow up, 3084 (41.4%) children had a birth interval of 24–47 months, only 2308 (31%)mothers of children had health facility delivery and 2579 (34.6%) had anemia.

### Factors associated with stunting

To see the selected maternal characteristics on childhood stunting, generalized estimating equation (GEE) with a Binary Logistic Regression function was done. Child sex, child age, residence, region, mother’s education, family size, source of drinking water, toilet facility, wealth index, size of child at birth, birth order, mother’s nutritional status, mother’s anemia, place of delivery were the factors showed significant association with stunting on the basis of p – value less than 0.2 in the bi-variable analysis.

By controlling all other variables (Table 3), the result of the final multivariable analysis revealed that, mother’s education status, mother’s nutritional status, and mother’s anemia were significantly associated with stunting.

**Table 3:** Factors associated with stunting among 6–59 months children in Ethiopia, May 2018 (*n* = 7452)

Variables	Stunting		COR (95% CI)	AOR (95%CI)
	Yes (%)	No (%)		
Sex of child				
Male	1571 (41.2)	2245 (58.8)	1.15 (1.05, 1.26)	1.20 (1.09, 1.33)*
Female	1389 (38.2)	2247 (61.8)	1.00	1.00
Age of child in months				
6–11	131 (14.8)	756 (85.2)	1.00	1.00
12–23	656 (38.8)	1036 (61.2)	3.64 (2.95, 4.49)	3.84 (3.10, 4.76)*
24–35	783 (48.3)	837 (51.7)	5.39 (4.37, 6.64)	5.76 (4.64,7.13)*
36–47	749 (47.3)	835 (52.7)	5.13 (4.16, 6.33)	5.35 (4.31, 6.64)*
48–59	641 (38.4)	1028 (61.6)	3.57 (2.90, 4.41)	3.68 (2.96, 4.57)*
Residence				
Urban	346 (26.8)	945 (73.2)	1.00	1.00
Rural	2614 (42.4)	3547 (57.6)	2.11 (1.78, 2.49)	0.85 (0.67, 1.06)
Region				
Tigray	352 (43.3)	461 (56.7)	3.88 (2.64, 5.70)	1.98 (1.30, 3.02)*
Afar	340 (48.6)	360 (51.4)	4.91 (3.32, 7.26)	1.75 (1.13, 2.73)*
Amhara	392 (50.8)	379 (49.2)	5.22 (3.56, 7.67)	2.56 (1.66, 3.94)*
Oromia	449 (38.3)	722 (61.7)	3.05 (2.09, 4.44)	1.50 (0.98,2.30)
Somali	274 (29.2)	665 (70.8)	2.10 (1.42, 3.09)	0.91 (0.60, 1.40)
Benshangul	300 (47.3)	334 (52.7)	4.41 (2.97, 6.56)	2.10(1.34, 3.28)*
SNNPR	398 (41.5)	560 (58.5)	3.54(2.42, 5.17)	1.87 (1.22, 2.86)*
Gambela	134 (28.1)	343 (71.9)	2.01 (1.32, 3.05)	0.94 (0.60, 1.48)
Harari	118 (34.4)	225 (65.6)	2.48 (1.60, 3.84)	1.62 (1.02, 2.57)*
Dire Dawa	154 (44.9)	189 (55.1)	3.63 (2.35, 5.59)	2.11 (1.34, 3.32)*
Addis Ababa	49 (16.2)	254 (83.8)	1.00	1.00
Mother’s educational status				
No education	2093 (43.4)	2730 (56.6)	2.36 (1.95, 2.86)	1.51 (1.19, 1.91)*
Primary	712 (37.2)	1203 (62.8)	1.84 (1.50, 2.25)	1.42 (1.13, 1.78)*
Secondary /above	155 (21.7)	559 (78.3)	1.00	1.00
Family size				
2–4	738 (36.70	1271 (63.3)	1.00	1.00
5–7	1511 (41.4)	2139 (58.6)	1.21 (1.08,1.35)	1.11 (0.96,1.27)
> =8	711 (39.7)	1082 (60.3)	1.14 (1.00, 1.31)	1.0 (0.84, 1.20)
Source of drinking water				
Improved water source	1718 (38.4)	2754 (61.6)	1.00	1.00
Unimproved water source	1242 (41.7)	1738 (58.3)	1.13 (1.02, 1.26)	0.91 (0.81,1.02)
Type of toilet facility				
Improved toilet	301 (25.4)	885 (74.6)	1.00	1.00
Unimproved toilet	1241 (40.7)	1810 (59.3)	1.77 (1.51, 2.08)	1. 29 (1.10, 1.60)*
Open defecation/bush/field	1418 (44.1)	1797 (55.9)	2.14 (1.82,2.69)	1. 32 (1.06, 1.58)*
Wealth index				
Poor	1810 (45.2)	2192 (54.8)	1.94 (1.72, 2.19)	1.59 (1.35, 1.87)*
Medium	454 (40.5)	667 (59.50	1.54 (1.31, 1.79)	1.27 (1.07,1.52)*
Rich	696 (29.9)	1633 (70.1)	1.00	1.00
Size of child at birth				
Large	770 (34.1)	1485 (65.9)	0.83(0.74, 0.93)	0.82 (0.73, 1.03)
Average	1264 (38.9)	1989 (61.1)	1.00	1.00
Small	926 (47.6)	1018 (52.4)	1.39 (1.24,1.56)	1.38 (1.22, 1.56)*
Birth order number				
1	529 (36.7)	914 (63.3)	1.00	1.00
2–3	887 (37.1)	1504 (62.9)	o.99(0.87,1.13)	0.92 (0.79, 1.07)
4–5	750 (42.8)	1001 (57.2)	1.24 (1.08, 1.44)	1.02 (0.85, 1.22)
> =6	794 (42.5)	1073 (57.5)	1.18 (1.03,1.37)	1.03 (0.85, 1.25)
Mother’s nutritional status				
Under weight	801 (43.40	1044 (56.6)	1.16 (1.04, 1.29)	1.20 (1.06, 1.35)*
Normal	2004 (40.3)	2963 (59.7)	1.00	1.00
Over weight/ Obese	155 (24.2)	485 (75.8)	0.57 (0.47, 0.68)	0.76 (0.61, 1.03)
Mother’s anemia status				
Not anemic	1875 (38.5)	2998 (61.5)	1.00	1.00
Anemic	1085 (42.1)	1494 (57.9)	1.16 (1.05, 1.29)	1.18 (1.06, 1.32)*
Place of delivery				
Health facility	728 (31.5)	1580 (68.5)	1.00	1.00
Home	2232 (43.4)	2912 (56.6)	1.57 (1.40, 1.75)	1.09 (0.95, 1.26)

NB: - * statistical significant variables at p < 0.05

Accordingly, children whose mothers had no education were 1.51 times (AOR = 1.51; 95%CI: 1.19, 1.91) more likely to be stunted as compared to children of mother who had educational status of secondary and above. Likewise, the odds of being stunted among children whose mothers had primary education were 1.42 times (AOR = 1.42; 95%CI: 1.13, 1.78) compared to children whose mother had higher educational status.

The finding of this study also identified that mother’s nutritional status had significant association with stunting. Children whose mothers had underweight nutritional status were1.20 times (AOR = 1.20; 95%CI: 1.06, 1.35) more likely to be stunted as compared to children of mothers with normal nutritional status.

Finally, the likelihood of being stunted was 1.18 times (AOR = 1.18; 95%CI: 1.06, 1.32) higher among children whose mothers had anemia compared to their counter parts (Table 3).

### Factors associated with wasting

We follow similar procedure to identify maternal characteristics associated with wasting. From the final model three variables, maternal nutritional status, birth interval and place of delivery were associated with wasting (Table 4).

**Table 4:** Factors associated with wasting among 6–59 months children, Ethiopia May 2018 (n = 7452)

Variables	wasting		COR (95% CI)	AOR (95%CI)
	Yes (%)	No (%)		
Sex of child				
Male	481 (12.6)	3335 (87.4)	1.18 (1.03,1.37)	1.26 (1.09,1.46)*
Female	392 (10.8)	3244 (89.2)	1.00	1.00
Age of child in months				
6–11	156 (17.6)	731 (82.4)	1.92(1.52,2.43)	1.95 (1.52,2.49)*
12–23	249 (14.7)	1443 (85.3)	1.56(1.27,1.92)	1.61 (1.29,2.00)*
24–35	171 (10.6)	1449 (89.4)	1.05(0.84,1.32)	1.05 (0.83, 1.32)
36–47	129 (8.1)	1455 (91.9)	0.76(0.60,0.97)	0.77 (0.60,0.98)*
48–59	168 (10.1)	1501 (89.9)	1.00	1.00
Residence				
Urban	123 (9.5)	1168 (90.5)	1.00	1.00
Rural	750 (12.2)	5411 (87.8)	1.54 (1.18,2.00)	0.65 (0.48, 1.09)
Region				
Tigray	93 (11.4)	720 (88.6)	1.00	1.00
Afar	128 (18.3)	572 (81.7)	1.81 (1.28,2.55)	1.37 (0.96, 1.96)
Amhara	71 (9.2)	700 (90.8)	0.80 (0.55,1.18)	0.78 (0.53, 1.16)
Oromia	118 (10.10	1053 (89.9)	0.86 (0.61,1.22)	0.91 (0.63, 1.30)
Somali	200 (21.3)	739 (78.7)	2.12 (1.54,2.92)	2.10 (1.50,2.94)*
Benshangul	67 (10.6)	567 (89.4)	0.92 (0.61,1.37)	1.04 (0.68, 1.56)
SNNPR	55 (5.7)	903 (94.3)	0.49 (0.32,0.74)	0.57 (0.37,0.87)*
Gambela	70 (14.7)	407 (85.3)	1.37 (0.93,2.03)	1.32 (0.89, 1.96)
Harari	33 (9.6)	310 (90.4)	0.83 (0.51,1.35)	1.02 (0.62, 1.67)
Dire Dawa	32 (9.3)	311 (90.7)	0.73 (0.44,1.23)	0.78 (0.47, 1.32)
Addis Ababa	6 (2.0)	297 (98.0)	0.17 (0.07,0.41)	0.28 (0.11,0.71)*
Mother’s educational status				
No education	641 (13.3)	4182 (86.7)	1.99 (1.45,2.73)	1.29 (0.89, 1.87)
Primary	183 (9.6)	1732 (90.4)	1.45 (1.04,2.03)	1.19 (0.82, 1.71)
Secondary / above	49 (6.9)	665 (93.1)	1.00	1.00
Family size				
2–4	200 (10.0)	1809 (90.0)	1.00	1.00
5–7	444 (12.2)	3206 (87.8)	1.22 (1.02,1.46)	1.20 (0.97, 1.48)
> =8	229 (12.8)	1564 (87.2)	1.23 (1.0, 1.52)	1.08 (0.83, 1.42)
Type of toilet facility				
Improved toilet	106 (8.9)	1080 (91.1)	1.00	1.00
Unimproved toilet	285 (9.3)	2766 (90.7)	1.16 (0.90,1.50)	1.14 (0.86, 1.51)
Open defecation/bush/field	482 (15.0)	2733 (85.0)	1.79 (1.40,2.29)	1.22 (0.92, 1.62)
Wealth index				
Poor	579 (14.5)	3423 (85.5)	1.94 (1.60,2.36)	1.34 (1.03,1.74)*
Medium	117 (10.4)	1004 (89.6)	1.47 (1.14,1.89)	1.29 (0.97, 1.72)
Rich	177 (7.6)	2152 (92.4)	1.00	1.00
Size of child at birth				
Large	201 (8.9)	2054 (91.1)	0.79(0.66, 0.95)	0.80(0.66, 1.06)
Average	357 (11.0)	2896 (89.0)	1.00	1.00
Small	315 (16.2)	1629 (83.8)	1.55 (1.31,1.83)	1.44 (1.21,1.72)*
Birth order number				
1	147 (10.2)	1296 (89.8)	1.00	1.00
2–3	244 (10.2)	2147 (89.8)	1.00 (0.80,1.24)	0.91 (0.72, 1.15)
4–5	228 (13.0)	1523 (87.0)	1.32 (1.05,1.64)	1.08 (0.82, 1.42)
> =6	254 (13.6)	1613 (86.4)	1.37 (1.10,1.71)	1.16 (0.87, 1.55)
Preceding birth interval				
First birth	166 (11.5)	1277 (88.5)	1.04 (0.82,1.31)	1.04 (0.82, 1.30)
< 24	212 (14.0)	1303 (86.0)	1.28 (1.02,1.59)	1.31 (1.04,1.64)*
24–47	334 (10.8)	2750 (89.2)	0.95 (0.78,1.17)	0.95 (0.77, 1.12)
> =48	161 (11.4)	1249 (88.6)	1.00	1.00
Diarrhea in last two weeks				
No	745 (11.4)	5808 (88.6)	1.00	1.00
Yes	128 (14.2)	771 (85.8)	1.32 (1.08,1.62)	1.14 (0.91, 1.43)
Fever in last two weeks				
No	715 (11.3)	5633 (88.7)	1.00	1.00
Yes	158 (14.3)	946 (85.7)	1.31 (1.09,1.58)	1.17 (0.95, 1.45)
Mother’s nutritional status				
Under weight	318 (17.2)	1527 (82.8)	1.68 (1.44,1.97)	1.52 (1.29,1.79)*
Normal	513(10.3)	4454 (89.7)	1.00	1.00
Over weight/ Obese	42 (6.6)	598 (93.4)	0.61(0.44, 0.80)	0.67 (0.48, 1.05)
Place of delivery				
Health facility	199 (8.6)	2109 (91.4)	1.00	1.00
Home	674 (13.1)	4470 (86.9)	1.48 (1.24,1.77)	1.24 (1.04,1.52)*

NB: - * statistical significant variables at p < 0.05

Based on the results, children whose mothers had underweight nutritional status were 1.52 times (AOR = 1.52; 95%CI: 1.29, 1.79) more likely to be wasted as compared to children of mothers who had normal nutritional status.

The likelihood of developing wasting was 1.31 times (AOR = 1.31; 95%CI: 1.04, 1.64) higher among children of birth interval less than 24 months as compared to children of birth interval greater or equal to 48 months.

As compared to children whose mothers had health facility delivery, children of mothers with home delivery were1.24 times (AOR = 1.24; 95%CI: 1.04, 1.52) more likely to be wasted.

## Discussion

Recognizing under nutrition among children is vital since it affects the health and long term productivity of the child [1]. Regarding maternal characteristics associated with stunting and wasting, analysis of this study indicated that mother’s educational status, mother’s nutritional status, and mother’s anemia status were significantly associated with stunting. Similarly, mother’s nutritional status, preceding birth interval and place of delivery were significantly associated with wasting.

Mother’s educational status was significantly associated with stunting. Children whose mothers had no education were more likely to be stunted as compared to children whose mother had educational status of secondary or above. Also children whose mothers had primary education were higher risk of being stunted. This finding is consistent with the study conducted in Tanzania [16], Kenya [17], and Ethiopia [1, 18, 19]. This might be due to mother who had no education had limited knowledge which related to better child feeding and caring, low income and low living conditions [20]. Education of women has several positive effects on the quality of care rendered to children since women are the main care takers of children. Their ability to process information, acquire skills, and positive caring behavior improves with education. Educated women use health care facilities, interact more effectively with health professionals, comply with treatment recommendations, and keep their environment clean. Also, more educated mothers are committed to child care and interact very well with their children [19]. Moreover, education of mothers improves child health by altering intra-household allocation of resources in a manner that favors children. Educated mothers are more likely to follow child feeding recommendations, which ultimately improves dietary diversity and meal frequency and nutritional status [21].

The current study found that children of underweight mothers are more likely to be stunted than children of normal weight mothers. This finding is supported by studies conducted in Ethiopia [22], Nigeria [23], Tanzania [24],and Brazil [25]. It is known that malnutrition has an intergenerational cycle of malnutrition. As a result, maternal deficiency means infant deficiency and a risk factor for fetal growth restriction, result in low birth weight [25, 26]. Low birth weight means more depletion of already low stores of key growth nutrients, which the mother’s breast milk continues to lack due to poor maternal nutritional status, thus resulting in a prolonged lack in these children [27].

This study also showed that children of anemic mothers are more likely to be stunted. This might be children of anemic mother’s influences fetal growth and birth weight [28]. This results more depletion of already low stores of key growth nutrients. There is ample evidence supporting the fact that stunting begins in utero as a result of trans-generational relationship, and anemia is a strong predictor of stunting [29, 30].

This study showed that the level of wasting was also higher in children of underweight mothers as compared to children of normal weight mothers. This was also observed in Ethiopia [31], and Sub-Saharan Africa countries [32]. This could be explained by the presence of an intergenerational link between maternal and child nutrition means a small mother will have small babies who in turn grow to become small mothers [31, 33].

The findings of this study showed that preceding birth interval of children is a significant predictor of nutritional status. Children having birth interval less than 24 months had higher risk of being wasting as compared with children having greater than or equal to 48 month’s birth interval. This study was in line with the study conducted in Bangladesh [34, 35]. This might be due to short birth interval between birth might pose sharing problems among living siblings and parents can’t take better care of their children and compromise the breastfeeding duration of the index child [36]. The mother herself may be biologically depleted from too frequent births, and this could also negatively affect the nutritional status of the newborn baby as a result of the intergenerational link [1].

The findings of this study showed that place of delivery of mother is a significant predictor of nutritional status of children. Children whose mothers had home delivery were higher risk of being wasted than children whose mothers had health facility delivery. This finding in line with study in Ethiopia [31]. This might be due to information gap regarding child feeding practice due to their poor health care seeking behavior to [1].

### Limitations

The cross-sectional nature in this study, whereby it may not explain the temporal relationship between maternal characteristics and child nutritional status. Further, sample weighting was not considered in order to avoid over complexity of the generalized estimation equations model. There might be recall bias during dietary recall and answering other child characteristics. The mother’s social value was not available in the data set and not considered in the analysis.

## Conclusion

Maternal education, maternal nutritional status, and maternal anemia status were associated with stunting. Also maternal nutritional status, place of delivery, and preceding birth interval were associated with wasting. Therefore, there is needed to enhance the nutritional status of children by improving maternal nutritional status, maternal education, maternal anemia status and prolonging birth interval, and promoting health facility delivery.

## Abbreviations

ANC: Antenatal Care
AOR: Adjusted Odd Ratio
BMI: Body Mass Index
CI: Confidence Interval
COR: Crude Odd Ratio
CSA: Central Statistical Agency
EAs: Enumeration Areas
EDHS: Ethiopia Demographic and Health Survey
GEE: Generalized Estimating Equation
SD: Standard Deviation
SNNPR: South Nations and Nationality of Peoples Republic
SPSS: Statistical Package for Social Sciences
SSA: Sub Saharan Africa
UNICEF: United Nations Children’s Emergency Fund
USAID: United States Agency for International Development
WHO: World Health Organization
